# Supplementary material for: Metformin Inefficiency to Lower Lipids in Vitamin B12 Deficient HepG2 Cells Is Alleviated via Adiponectin-AMPK Axis
Source: Nutrients. 2023 Dec 8;15(24):5046. doi: 10.3390/nu15245046 (PMC10745523; doi:10.3390/nu15245046)
Supplement: Supplementary file 1 [file nutrients-15-05046-s001.zip › nutrients-2705943-supplementary.pdf]

Supplementary material

Experimental procedure

Hep G2 source and authenticity:

Hep G2 cell line (ATCC, Manassas, Virginia, USA) is mainly an epithelial, continuous cell line which is characteristically adherent to cell culture plates. The cells were originally developed subsequent to isolation from liver biopsy of a 15-year-old male Caucasian American who was diagnosed with a well-differentiated hepatocellular carcinoma [56, 57]. Under suitable conditions of culture environment, the Hep G2 model is highly suitable for *in vitro* experiments targeting the examination of metabolism of lipids in the liver of humans as a result of its capacity to grow on a large scale and furthermore demonstrating a high level of differentiation both physiologically and morphologically *in vitro*. All cell cultures in this study were therefore done using the Hep G2 cell line.

Hep G2 cells, used throughout the experiments, were formerly cryopreserved within cryovials and stored in the liquid nitrogen. The cells were thawed at 37ºC using the water bath. The resultant suspension of cells was steadily added to an initially warmed (37ºC) 10ml EMEM media in a clean and sterile 50ml falcon tube, followed by centrifugation at 250g for 5 minutes. The supernatant was then discarded whereas the pellet of cells was carefully suspended in 20ml of fresh pre-warmed (37ºC) custom-made B12-deficient-EMEM medium supplemented with 10% FBS, 1% L-glutamine and 1% penicillin/ streptomycin in a 50ml falcon tube. The entire suspension of cells was then transferred into a sterile T75 ($75cm^2$) cell/tissue culture flask (Sigma, UK) and incubated at 37ºC at 5% CO_2 saturation, with routine changing of media every 48-hours until reaching confluence.

Mycoplasma test

Before utilizing HepG2 cells for the current experiment, the cells were tested for mycoplasma contamination as similarly described elsewhere [58]. To assess the presence of mycoplasma contamination in cell culture, briefly, we employed an indirect detection method in the cell culture. Test cells were initially grown for a maximum of three passages and finally cultured in an antibiotic-free EMEM medium until 80-100% confluence in a 6-well plate. Concurrently, reporter cells were carefully seeded in a similar antibiotic-free medium using a sterile cover-glass into the base of the 6-well plate. About 1000 µl of the medium from test cells was pipetted unto the reporter cells and incubated for 72 hours at 37 ºC and 5% CO_2 saturation. The medium

was carefully removed and cells rinsed with PBS, followed by 5 minutes fixation of cells with 1:3 acetic acid-methanol mixture. After discarding the fixative, cells were washed and stained at room temperature with Hoechst 33258 for 15 minutes and observed using UV microscope.

References

55. Aden, D.P., et al., Controlled synthesis of HBsAg in a differentiated human liver carcinoma-derived cell line. *Nature.* **1979**, 282(5739): p. 615-616
56. López-Terrada, D., Cheung, S.W., Finegold, M.J. and Knowles, B.B. Hep G2 is a hepatoblastoma-derived cell line. *Human pathology.* **2009**. 40(10): p. 1512.
57. Young, L., Sung, J., Stacey, G., & Masters, J. R.. Detection of Mycoplasma in cell cultures. *Nature protocols*, **2010**. **5**(5): p. 929-934.
